# A Comparison of Clinicopathological Features and Molecular Markers in British and Nigerian Women with Breast Cancer

**DOI:** 10.4137/cmo.s474

**Published:** 2008-04-24

**Authors:** Isaac D. Gukas, Anne C. Girling, Barnabas. M. Mandong, Wendy Prime, Barbara A. Jennings, Samuel J. Leinster

**Affiliations:** 1 School of Medicine, Health Policy and Practice, University of East Anglia, Norwich, NR47TJ, U.K; 2 Department of Histopathology, Norfolk and Norwich University Hospital, Colney lane, Norwich; 3 Pathology, Jos University Teaching Hospital, Jos, PMB 2076 Jos, Nigeria; 4 Cancer Tissue Bank Research Centre, University of Liverpool, Liverpool, U.K

**Keywords:** breast cancer, nigerian, immunocytochemistry, molecular markers

## Abstract

**Background:**

Some studies have suggested that breast cancer in black women is more aggressive than in white women. This study’s aim was to look for evidence of differences in tumour biology between the two cohorts.

**Methods:**

This study compared the stage, grade and pathological expression of five immunohistochemical markers (oestrogen receptor [ER], progesterone receptor [PR], ERBB2, P53 and cyclin D1 [CCND1]) in tumour biopsies from age-matched cohorts of patients from Nigeria and England. Sixty-eight suitable samples from Nigerian (n = 34) and British (n = 34) breast cancer patients were retrieved from histology tissue banks.

**Results:**

There were significant differences between the two cohorts in the expression of ER and CCND1; and stark differences in the clinical stage at presentation. But no significant differences were observed for tumour grade.

**Conclusion:**

There was a significantly, low ER expression in the Nigerian cases which also predicts a poor response to hormonal therapy as well as a poorer prognosis. Differences in clinical stage at presentation will most likely influence prognosis between Nigerian and British women with breast cancer.

## Introduction

There is significant worldwide variation in the incidence and mortality from breast cancer. (Key et al. 2001) Studies carried out in the United States among white and black women have found that black women have a lower lifetime risk of breast cancer but have poorer survival rates following diagnosis. (Harris et al. 1992; Elledge et al. 1994) The same trends can be seen between Nigerian and English populations in the reported incidence of symptomatic breast cancer and subsequent five year survival. (Ihekwaba, 1992; Ihezue CH et al. 1994; Quinn M and Allen E, 1995; Okobia and Osime, 2001)

Many factors could explain differences in reported disease incidence and mortality. Demographic variation such as life expectancy will affect breast cancer incidence rates (Gukas ID et al. 2006) and socioeconomic differences affect awareness of breast cancer, access to healthcare (including screening) and reporting rates through cancer registries. (Harris et al. 1992; Ihekwaba, 1992; Gwyn et al. 2004)

However, environmental and/or genetic factors could lead to differences in tumour biology; such differences may result in differential response to treatment. Elledge et al. showed that black Americans were less likely to have tumours with positive ER and PR status (Elledge et al. 1994) and had poorer survival than white Americans.

In this study we have compared the tumour type, grade, disease stage and the expression of a panel of immunocytochemical markers in age-matched cohorts of breast cancer patients from Nigeria, a predominantly black population (over 99.9%) (National Population Commission of Nigeria. 1998), and England, within a predominantly white population (over 97% in Liverpool and 98% in East Anglia) (U.K. National Statistics,). Our aim was to look for evidence of differences in tumour biology between the two cohorts.

## Methods

### Ethics and research governance

This study had approval from the ethics committee of Jos University Hospital, Plateau State, Nigeria and from the custodians of the Royal Liverpool and Broadgreen University tissue bank; the Norfolk and Norwich University Hospital research governance and local research ethics committees have also approved this study (LREC 2002–3/049).

### Histology and TNM staging

46 Paraffin-wax embedded tissue biopsies were retrieved from the histopathology archives of Jos University Teaching Hospital, Nigeria; these were from a consecutive series of breast cancer patients diagnosed between 1999 and 2001. Analysis of haematoxylin and eosin stained sections from these blocks was used to determine if there was adequate tumour material available for subsequent tumour grading and immunocytochemistry. 34 cases were considered suitable. The clinical staging information, using the tumour node metastasis (TNM) system, was obtained for these cases based on physical examination and palpation. Pathological staging is not routinely carried out in Nigeria because of limited healthcare resources.

The 34 Nigerian cases were paired with age-matched British breast cancer cases held at tissue banks in Norwich (n = 14) and Liverpool (n = 20). Paraffin wax embedded tissue biopsies were retrieved for these cases along with pathological staging data, using the TNM system.

The 68 tumours were typed and graded histologically according to national guidelines. (Royal College of Pathologists, 2005)

### Immunocytochemistry

Immunocytochemical staining of tissue sections from paraffin wax embedded biopsies was performed within the diagnostic laboratories of the histopathology department at Addenbrooke’s hospital, Cambridge. Antigen retrieval was achieved by microwave oven incubation for five minutes in citrated buffer (0.01M: pH 6.0) testing for ER, PGR, ERBB2 and p53 oncoproteins and in 1mM EDTA (pH 8.0) for CCND1. Antigen localisation was achieved by incubating sections with primary antibodies at various dilutions.

Sections were incubated with the antibodies ERBB2 (Dako, Glostrup, Denmark) at a dilution of 1:1000, ER (6F11; Novacastra, Newcastle, U.K.) at a dilution of 1:15, PGR (PgR 636; Carpinteria, U.S.A.) at a dilution of 1:50, P53 (DO-7; DAKO Glostrup, Denmark) at a dilution of 1:50 and CCND1 (P2D11F11; Novacastra, Newcastle, U.K.) at a dilution of 1:20. The indirect avidin-biotin complex (ABC) procedure was applied for detection of the bound antibody. Scoring was carried out by two independent scorers (IG and AG). Nuclear staining of at least 10% of malignant cells was considered positive for ER, PR, Cyclin D1 and P53. Cell membrane staining of at least 5% was used as a cut off for ERBB2 positive staining.

### Statistics

The Chi square or Fisher’s exact test with Yate’s correction was used to compare the data from the two cohorts.

## Results

### Histology and TNM staging

The mean age of the cohort of 68 patients was 45 (median age 43 years, and age range 26–81 years).

Analysis of tumour type showed that 33 of the Nigerian cases were invasive ductal carcinomas of no special type (NST). One tumour was poorly differentiated and showed spindle cell/sarcomatoid features. There were no carcinomas of invasive lobular type. In the British cohort, 31 cases were invasive ductal carcinomas NST. There were two cases of mixed type (showing both ductal and lobular features) and one medullary carcinoma.

The tumour types and grades of these 68 tumour are shown in tables 1 and 2. Ten high power fields were examined to grade the tumour. For two of the (grade 2) Nigerian cases, the amount of tumour present was small and there was extensive necrosis, precluding the analysis of 10 high power fields; therefore these two cases may have been underscored for mitoses and thus grade. There were no significant differences in the grades of the age matched tumour in the two cohorts, regardless of the scoring (as grade 2 or 3) of the two Nigerian cases discussed above.

There were differences in the TNM staging data for the two cohorts (see Table 1) 61.8% of the Nigerian tumours being TNM stage 3 or 4 compared with 11.8% of the British tumour.

### Immunocytochemistry

The number of cases showing expression of ER and CCND1 was significantly higher in the British cohort than in the Nigerian cohort. Other markers showed no significant differences. The results of the statistical analyses for these 68 cases are shown in Table 2.

We have also compared the expression of the different molecular markers seen in clinical stages 1&2 (early stage) and clinical stages 3&4 (late stage) between U.K. cases and the Nigeria cases, as shown in Table 3. Only ER expression showed significant difference.

## Discussion

In this study we compared the histological and immunocytochemical profiles of age-matched cohorts of Nigerian and British breast cancer cases. The average age of the cohort of 68 cases was determined by the cases selected in Nigeria and was young (mean age = 45.2) relative to a typical series of British cases. (Gukas ID et al. 2006) The incidence of special type carcinomas was also low relative to other series. (Simpson and Page, 1994)

There were no significant differences between the cohorts for tumour grade. Immunocytochemical studies showed no significant differences between the cohorts in the cases expressing the poor prognostic markers P53 and ERBB2. The number of cases positive for ER and CCND1 was significantly higher in the cohort of British cases. A large study by Elledge et al. showed that breast tumours are more likely to be ER negative in black than white Americans but showed no differences in the expression of P53 or ERBB2. (Elledge et al. 1994)

An inverse relationship between ER and PR expression and high tumour grade has been previously documented (Millis, 1980) and was also noted for the Nigerian tumours (p = 0.006 for ER and p = 0.002 for PR) but there was a less significant trend for the British tumours (p = 0.053 for ER and p = 0.014 for PR). It has been suggested that, as part of the natural history of breast cancer, some tumours tend to lose hormone receptor status by progressive genetic alterations (Stoner et al. 2002). The difference in receptor status between the two groups could therefore be explained by the late stage of the Nigerian cases. This is supported by our finding of consistent lack of expression of ER in the Nigerian tumours when early and late stage tumours were compared in the two countries (p = 0.0477 and p = 0.0261 respectively). The Nigerian tumours were close to the upper size limit for each stage. Our finding that tumour grade remained the same for both populations whereas stage and hormone status were worse for the Nigerians may support the notion that tumour grade does not deteriorate longitudinally. (Millis et al. 1998)

Other studies have shown that tumours that express hormone receptors are also more likely to express CCND1 (Shoker et al. 2001). This correlation was also seen for the British tumours (p = 0.004 for ER and p = 0.001 for PR) but there was no significant correlation for the Nigerian tumours (p = 0.068 for ER and p = 0.085 for PR).

21 of the 34 Nigerian cases presented with TNM stages 3 and 4. Pre-operative clinical staging was used for these cases which may incorrectly stage the tumour when compared with the gold standard of pathological staging. (Bosch et al. 2003) However, our data concur with other studies of Nigerian cohorts showing that patients commonly present with very large primary breast tumour and matted palpable axillary nodes and consequent high TNM stage. (Ihekwaba, 1992; Ihezue CH et al. 1994; Okobia and Osime, 2001) The risk of over-staging is low when tumours are close to the upper size limit for a particular stage. (Benson et al. 2003) Therefore, despite the limitation described with the use of different staging methods, we are confident that there are clear differences in TNM staging between the two cohorts; stages 3 and 4 being more common for the Nigerian than the British cases (odds ratio = 12.1, confidence interval = 3.5 to 42.4).

## Conclusions

There were significant differences in hormone receptor levels. This will predict poorer response to hormonal therapy and overall poorer prognosis in the Nigerian patients compared to the British patients. The difference observed in the frequency of CCND1 expression is an interesting finding, warranting further investigation. The late stage at presentation in the Nigerian cases could partly explain the difference in breast cancer prognosis for Nigerian and British women. There is a need for a stage for stage comparison to further study this subset of tumours.

These conclusions should now be tested in a large prospective case—controlled study.

## Figures and Tables

**Table 1:** The histological types and staging data for 68 British and Nigerian tumors.

	Invasive ductal NST	Other	TNM stage 1	TNM stage 2	TNM stage 3	TNM stage 4
Nigerian tumor (n = 34)	33	1*	0 (0%)	13 (38.2%)	14 (41.2%)	7 (20.6%)
UK tumor (n = 34)	31	3†	5 (14.7%)	25 (73.5%)	4 (11.8%)	0 (0%)

The cases were staged using the UICC TNM system; preoperative clinical staging is shown for the Nigerian cohort; pathological staging was used for the British cohort.

* Poorly differentiated carcinomas with sarcomatoid features.

† Two cases were of mixed type (showing both ductal and lobular features) and one case was a medullary carcinoma.

**Table 2:** The immunocytochemical and grading data for 68 British and Nigerian tumor.

	UK samples positive/total (%)	Nigerian samples positive/total (%)	p value
**Molecular markers**			
ER	20/34 (58.8)	9/34 (26.5)	p = 0.0142
PR	18/34 (52.9)	10/34 (29.4)	p = 0.0846
ERBB2	15/34 (44.1)	8/34 (23.5)	p = 0.1241
CCND1	9/33 (27.3)	2/33 (6.1)	p = 0.0475
P53	13/34 (38.2)	17/34 (50)	p = 0.4637
**Histological Grade**			
Grade 1	1/34 (2.9)	2/34 (5)	p = 1 (Fisher’s exact)
Grade 2	12/34 (35.3)	8/34 (23.5)	p = 0.4246
Grade 3	21/34 (61.8)	24/34 (70.6)	p = 0.6082

The result of grading and immunohistochemistry for ER, PR, ERBB2, P53 and CCND1 for the age-matched samples. There were significant differences for ER and CCND1 expression only (p < 0.05).

**Table 3:** Association between the molecular markers, and stage shown by p-value.

	Stage	UK cases	Nigerian cases	p-value
**ER**	1 and 2	16/30	2/13	p = 0.0477**
	3 and 4	4/4	7/21	p = 0.0261*
PR	1 and 2	14/30	2/13	p = 0.0855*
	3 and 4	2/4	8/21	p = 1*
ERBB2	1 and 2	14/30	2/13	p = 0.0855*
	3 and 4	1/4	6/21	p = 1*
**Cyclin D1**	1 and 2	7/29	0/13	p = 0.0791*
	3 and 4	2/4	2/20	p = 0.1149*
P53	1 and 2	11/30	8/13	p = 0.2404**
	3 and 4	2/4	9/21	p = 1*

Molecular marker expression has been presented according to stage. Early (stages 1 & 2) and late (stages 3 & 4) are compared in the two countries. Only ER expression showed significant difference.

* Fisher’s Exact Test.

** Chi-Square Test.

## Abbreviations

ER: oestrogen receptor
PR: progesterone receptor
CCND1: cyclin D1
TNM: tumour node metastasis
NST: no special type
